# The use of a machine-learning algorithm that predicts hypotension during surgery in combination with personalized treatment guidance: study protocol for a randomized clinical trial

**DOI:** 10.1186/s13063-019-3637-4

**Published:** 2019-10-11

**Authors:** M. Wijnberge, J. Schenk, L. E. Terwindt, M. P. Mulder, M. W. Hollmann, A. P. Vlaar, D. P. Veelo, B. F. Geerts

**Affiliations:** 1 Department of Anesthesiology, Amsterdam UMC, location Academic Medical Center, University of Amsterdam, Meibergdreef 9, Postbus 22660, 1105 AZ Amsterdam, The Netherlands; 2 Department of Intensive Care Medicine, Amsterdam UMC, location Academic Medical Center, University of Amsterdam, Meibergdreef 9, Postbus 22660, 1105 AZ Amsterdam, The Netherlands; 3 Department of Technical Medicine, University of Twente, Drienerlolaan 5, 7522 NB Enschede, The Netherlands

**Keywords:** Artificial intelligence, Blood pressure, Perioperative care, Anesthesiology, Hemodynamics

## Abstract

**Background:**

Intraoperative hypotension is associated with increased morbidity and mortality. Current treatment is mostly reactive. The Hypotension Prediction Index (HPI) algorithm is able to predict hypotension minutes before the blood pressure actually decreases. Internal and external validation of this algorithm has shown good sensitivity and specificity. We hypothesize that the use of this algorithm in combination with a personalized treatment protocol will reduce the time weighted average (TWA) in hypotension during surgery spent in hypotension intraoperatively.

**Methods/design:**

We aim to include 100 adult patients undergoing non-cardiac surgery with an anticipated duration of more than 2 h, necessitating the use of an arterial line, and an intraoperatively targeted mean arterial pressure (MAP) of > 65 mmHg. This study is divided into two parts; in phase A baseline TWA data from 40 patients will be collected prospectively. A device (HemoSphere) with HPI software will be connected but fully covered. Phase B is designed as a single-center, randomized controlled trial were 60 patients will be randomized with computer-generated blocks of four, six or eight, with an allocation ratio of 1:1. In the intervention arm the HemoSphere with HPI will be used to guide treatment; in the control arm the HemoSphere with HPI software will be connected but fully covered. The primary outcome is the TWA in hypotension during surgery.

**Discussion:**

The aim of this trial is to explore whether the use of a machine-learning algorithm intraoperatively can result in less hypotension. To test this, the treating anesthesiologist will need to change treatment behavior from reactive to proactive.

**Trial registration:**

This trial has been registered with the NIH, U.S. National Library of Medicine at ClinicalTrials.gov, ID: NCT03376347. The trial was submitted on 4 November 2017 and accepted for registration on 18 December 2017.

**Electronic supplementary material:**

The online version of this article (10.1186/s13063-019-3637-4) contains supplementary material, which is available to authorized users.

## Background

Worldwide, an estimated 313 million people have to undergo surgical procedures every year [1]. Intraoperatively, patients often suffer from episodes of hypotension. Hypotension, defined as a mean arterial pressure (MAP) < 65 mmHg, occurs in 65% of surgeries [2]. Intraoperative hypotension is usually caused by anesthetics, pre-operative use of medication, existing comorbidities or by the surgery itself [3].

Since both pressure and flow are required to deliver oxygen to the tissues, hypotension can negatively affect organ function [4]. Clinical cohort studies and one randomized controlled clinical trial have shown intraoperative hypotension to be associated with postoperative complications such as myocardial ischemia, renal insufficiency and increased mortality [5–11]. Not only the time spent in hypotension but also the severity (the depth) of hypotension may be important for postoperative outcome [12]. The time-weighted average (TWA) combines the time and depth of hypotension [13, 14].

Hypotension is most often preventable; however, current management of the hypotensive episodes is predominantly reactive and often occurs with some delay. Machine learning was used to develop an algorithm to predict hypotension minutes before the blood pressure actually decreases, the Hypotension Probability Indicator (HPI) [15]. The HPI algorithm is developed using continuously measured waveform data from 1334 patients, internally validated on a cohort of 350 patients and externally validated on a cohort of 204 patients. The HPI algorithm is able to predict hypotension with 88% sensitivity and 87% specificity minutes before a hypotensive event occurs [15].

We hypothesize that the use of the HPI algorithm in combination with a personalized treatment protocol will reduce the amount of time spent in hypotension measured by the TWA during non-cardiac surgery.

## Methods/design

### Study design

This investigator-initiated trial is divided into two phases. Phase A consists of prospective data collection in 40 patients to gain insight into the normal TWA in our study population. Phase-A data are collected to check our sample size for phase B and to verify if the control group is a representative sample. Phase B is a single-center, randomized controlled (1:1), superiority trial including 60 patients. The study takes place in the Academic Medical Center (AMC) Amsterdam, The Netherlands, a tertiary academic center. The study started with inclusion of the first patient in November 2017, the planned duration of the trial is 18 months. This trial has been registered with the NIH, U.S. National Library of Medicine at ClinicalTrials.gov (NCT03376347). This manuscript was written in accordance with the Standard Protocol Items: Recommendations for Interventional Trials (SPIRIT) guideline (Additional file 1) on reporting of intervention trial protocols [16].

### Eligibility criteria

Adult patients (aged 18 years or older) scheduled to undergo an elective, clinical, non-cardiac, surgical procedure under general anesthesia and requiring an arterial line will be eligible for inclusion. A desired target MAP of 65 mmHg during surgery is used as an inclusion criterion, to ensure that both study arms will be similar in this aspect. Patients undergoing emergency surgery are not eligible. Patients with cardiac failure, severe cardiac shunts, severe aortic stenosis and severe cardiac arrhythmias will be excluded in accordance with the summary of product characteristics of the HPI algorithm. Patients enduring significant hypotension before surgery and patients requiring dialysis will be excluded. Patients planned to undergo liver surgery or vascular surgery will be excluded because of the use of vascular clamping. For this trial, anesthesiologists are not allowed to use a different hemodynamics treatment protocol besides our study protocol; therefore, an exclusion criterion is the planned usage of a perioperative Goal Directed Fluid Therapy (GDFT) protocol.

Researchers will screen all patients presenting for elective, non-cardiac, non-day-case surgery. Patients will be contacted and informed in case of eligibility. Patient informed consent will be obtained the day prior to surgery.

### Study outline

Patients will be contacted on the surgical ward or at the pre-operative assessment clinic, and written information and oral explanation will be provided. Patient characteristics, medical history, medication use and American Society of Anesthesiologists (ASA) physical score classification will be collected from the medical records. Blood pressure measured at the outpatient clinic, blood pressure measured the day before surgery on the ward and blood pressure measured in the operating theater before induction will also be registered.

Phase A: TWA and normal treatment behavior of anesthesiologists in the AMC will be collected prospectively as baseline data. These data will be used to verify our sample size calculation for phase B and to study whether our control group is representative for the study population by comparing the baseline group versus the control group. During this phase of the study the treating anesthesiologist and anesthesia nurse will not be informed about the aim of the study or the endpoints measured.

Phase B: in this phase, patients will be randomized. The treating anesthesiologist and the anesthesia nurse will be informed about the study protocol and the usage of the HPI algorithm (with the secondary screen) the day before the surgery. All study interventions are to be performed by trained study personnel or the treating anesthesiologist, following instructions from the researchers.

In both study phases a researcher will be present – continuously – during all surgeries to note surgical and anesthetic details.

**Fig. 1:**
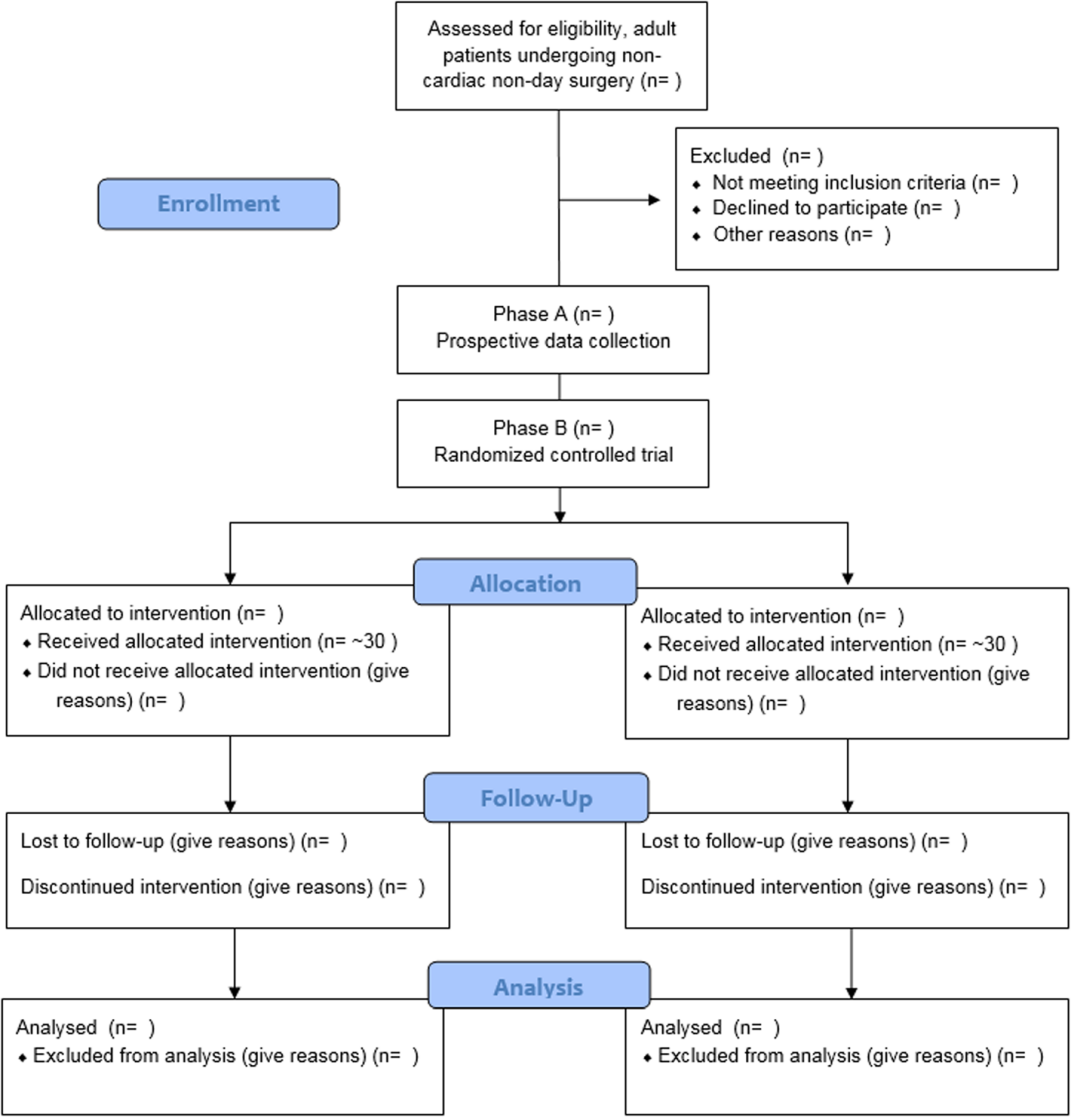
Consort Flow diagram

For a Consolidated Standards of Reporting Trials (CONSORT) flow diagram of the study see Fig. 1. All data will be entered using an electronic Clinical Report Form build in Castor EDC, a Good-Clinical-Practice-compliant data management system [17].

### Randomization and blinding

In phase B, patients will be randomized to either use of the HPI algorithm intraoperatively (intervention arm) or standard care (control arm). We will use a computer-generated, permutated block randomization, with a 1:1 allocation ratio. This will result in concealed and varying permutated block sizes of four, six or eight patients.

Randomization will be performed by a designated researcher. An independent researcher (not involved in collecting study data), blinded for the randomization, will perform the statistical analysis for the primary outcome.

### Study procedures and interventions

The HPI algorithm was previously internally and externally validated [15]. The HPI algorithm is only available on the HemoSphere and Flotrac monitoring systems and requires the use of a FlotracIQ sensor connected to an arterial line (Edwards Lifesciences Corp., Irvine, CA, USA). The FlotracIQ sensor has a splitter which enables the splitting of the arterial blood pressure signal to facilitate a blood pressure signal on both the Philips monitor (standard care) and the HemoSphere monitor (study).

**Fig. 2:**
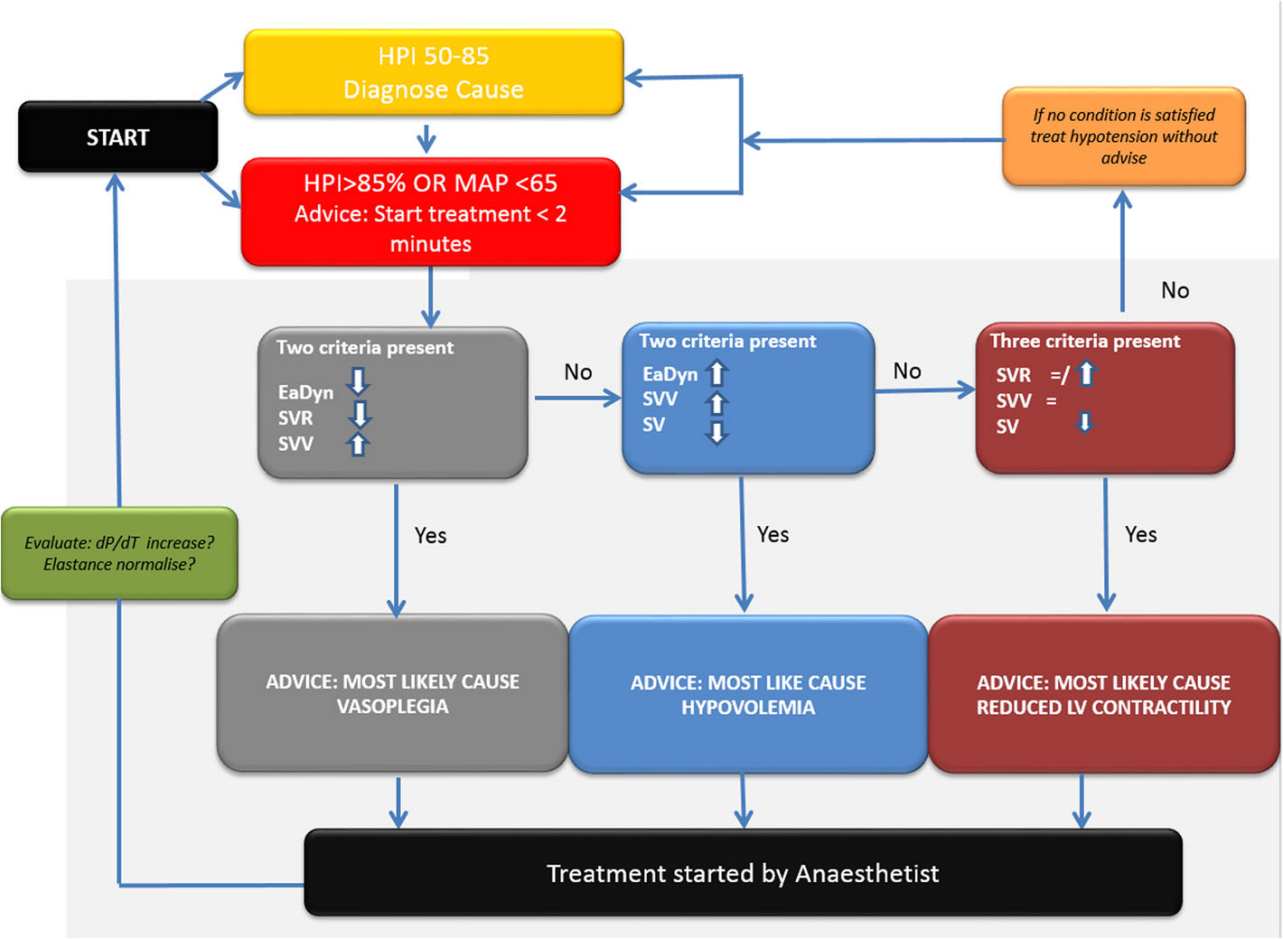
HYPE personalized treatment guidance protocol. HPI=hypotension prediction index. MAP= mean arterial pressure. EaDyn= dynamic arterial elastance. SVR= systemic vascular resistance. SVV= stroke volume variation. SV=stroke volume. dP/dT= delta pressure/delta time, measure for left ventricular function

**Fig. 3:**
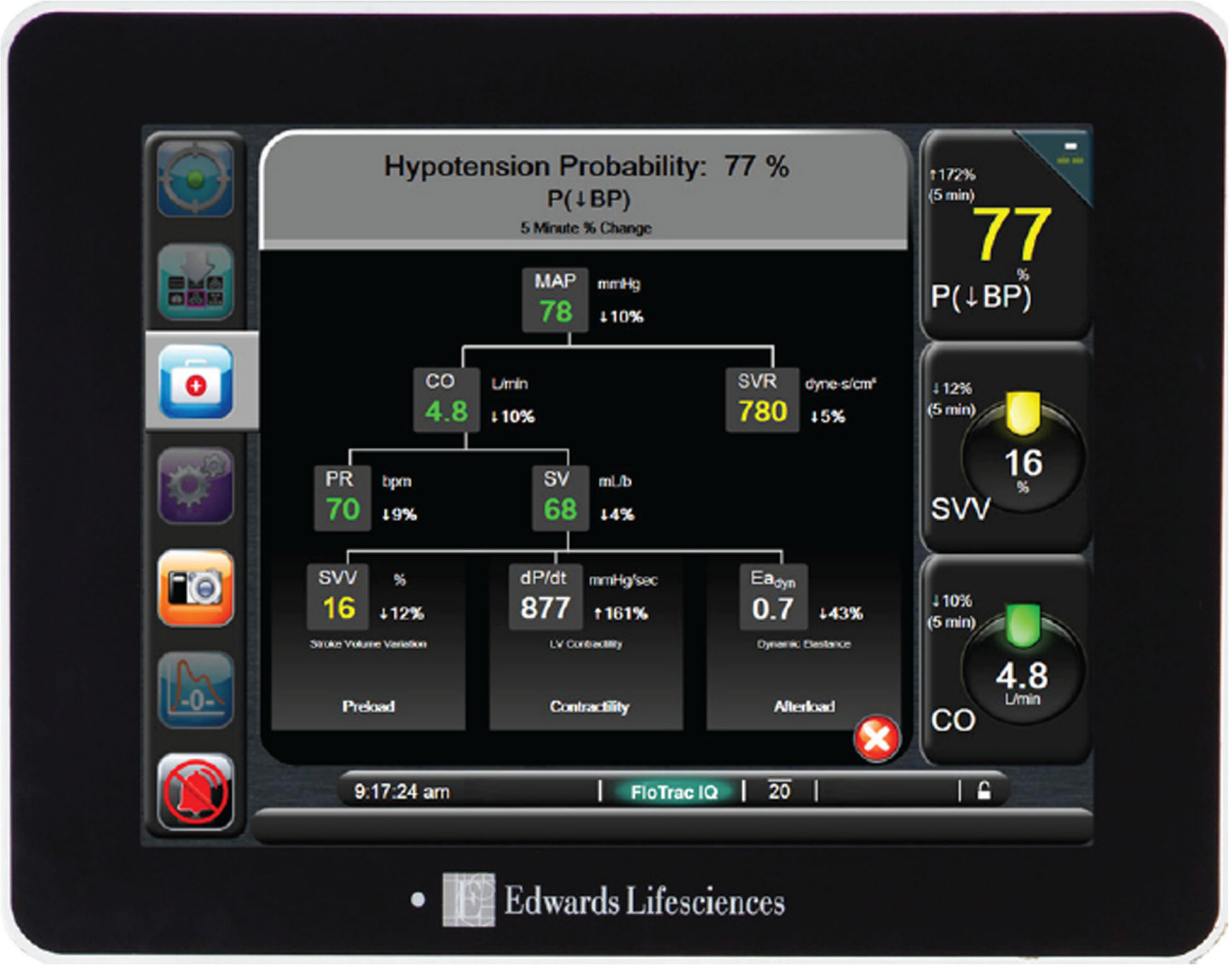
HemoSphere with HPI and secondary screen. P↓BP= probability of hypotension, this is a prediction ranging from 0-100%. MAP= mean arterial pressure. CO= cardiac output. SVR= systemic vascular resistance. PR= pulse rate. SV= stroke volume. SVV= stroke volume variation. dP/dt= delta pressure/delta time. Eadyn= dynamic arterial elastance

In all study participants this system will be connected to both the HemoSphere and the Philips monitor. The Philips monitor displays the MAP, systole, diastole and the pulse pressure variation as per standard care protocol in our hospital. In the baseline group (phase A) and in the control arm (phase B) the HemoSphere with HPI software will be connected; however, the screen will be fully covered. In the control arm the anesthesiologist solely uses the variables visible on the Philips monitor to guide hemodynamic treatment. In the intervention arm the HemoSphere with HPI software will be visible and the perioperative hemodynamic management will be based on both the Philips monitor and the HemoSphere monitor. Use of the HPI software is additional to standard care, it is not used as a replacement of standard care. In the intervention arm we will ask the anesthesiologist and anesthesia nurse to use the study treatment flowchart (Fig. 2). If the HPI alarm goes off, which entails both a sound and a flickering light, we ask the anesthesiologist to act upon this alarm preferably within 2 min. Use of the study treatment flowchart ensures that the anesthesiologist has to think about the underlying cause. The HemoSphere with HPI software has a second screen (Fig. 3) with variables that provide information about the underlying cause of the predicted hypotension (http://ht.edwards.com/scin/edwards/eu/sitecollectionimages/edwards/eu%20hpi%20brochure_digitalversion_largerfile%20for%20online%20use_10-6.pdf).

### Outcome measures

**Fig. 4:**
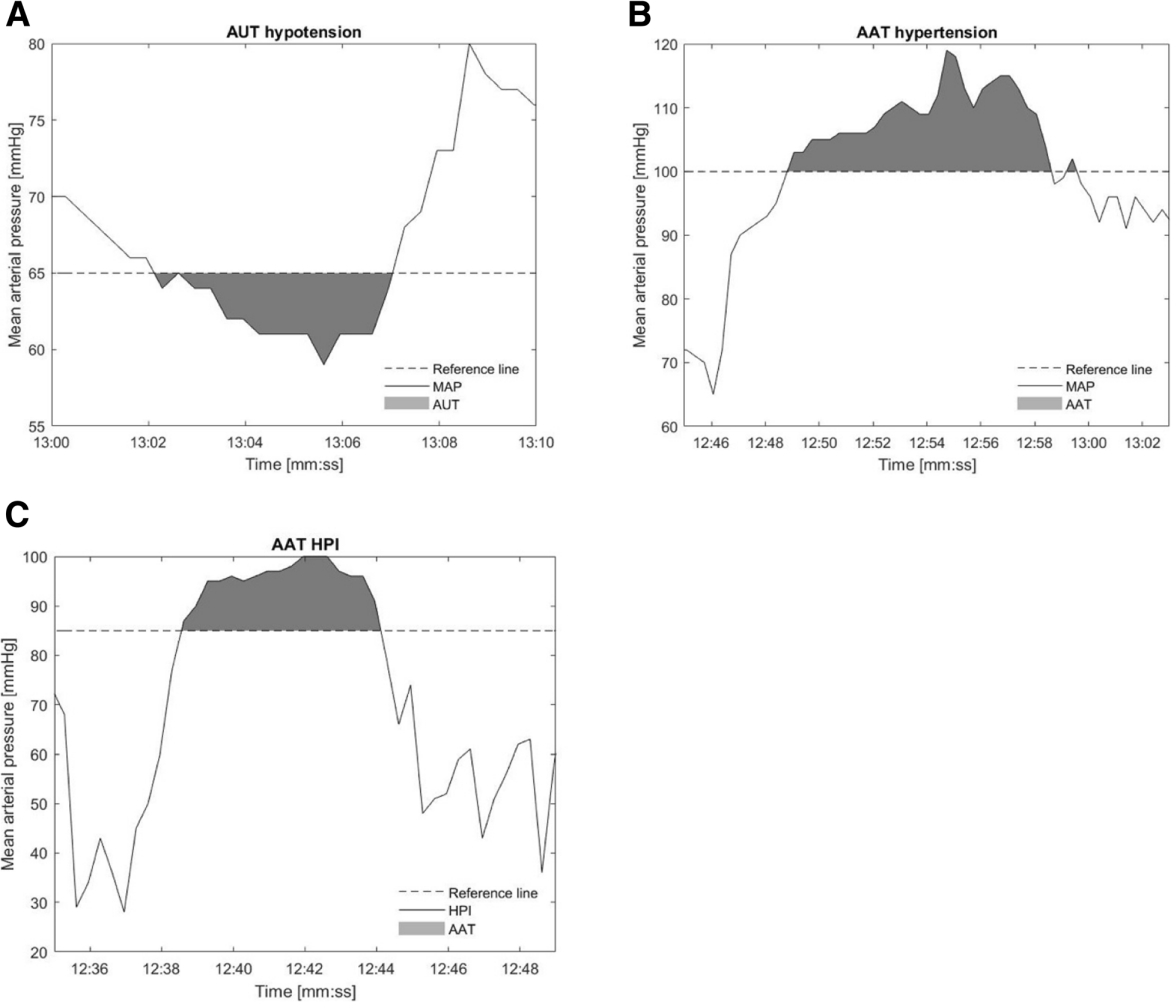
AUT and AAT calculations. **a** demonstrates the calculation of the area under (AUT) the curve used to calculate the TWA in hypotension. *TWA= (depth hypotension below MAP 65 threshold in mmHg x time spent below MAP 65 threshold in minutes, the AUT) / total duration operation in minutes)*. **b** and 4**c** demonstrate the calculation area above the curve (AAT) used to calculate the TWA in hypertension and the TWA of HPI alarm

Our primary outcome measure is the TWA in hypotension during surgery. The TWA is a calculation of the depth (in millimeters of mercury) of hypotension below the “threshold” MAP of 65 mmHg multiplied by the time spent in hypotension in minutes, this results in an area under the threshold AUT, see Fig. 4. To better compare this value between different operations this AUT will be divided by the total duration of the operation:

$$ \mathrm{Time}\ \mathrm{weighted}\ \mathrm{average}=\frac{\mathrm{depth}\ \mathrm{of}\ \mathrm{hypotension}\times \mathrm{time}\ \mathrm{spent}\ \mathrm{in}\ \mathrm{hypotension}}{\mathrm{total}\ \mathrm{surgery}\ \mathrm{time}} $$

Example: a MAP of 50 mmHg for 5 min results in an AUC of 75 (15 × 5). The total duration of the operation in minutes is 120 min. TWA = 75/120 = 0.625.

Hypotension is defined as a MAP < 65 mmHg for 1 min. An HPI alarm is defined as an HPI value of 85% and above during at least 1 min. A subsequent hypotensive episode, as well as an HPI alarm only counts as two separate events when respectively the MAP or the HPI will be normal for at least 1 min.

The secondary outcome measures include incidence of hypotension, time in hypotension, the percentage of time in hypotension and the AUC of a MAP < 65 mmHg. The above-mentioned parameters including TWA will also be assessed for hypertension (defined as MAP > 100 mmHg for at least 1 min) and for the HPI alarms. For hypertension and HPI alarm the area above the curve (AAT) will be calculated instead of the AUC, see Fig. 4. We will assess the treatment behavior of hypotension and HPI. This includes treatment choice (i.e., vasopressors, fluids, inotropes, position changes), treatment dose, time to treatment and feasibility of working with HPI based on the number of protocol violations.

Exploratory outcomes include underlying cause(s) of intraoperative hypotension and we will assess whether the use of HPI intraoperatively will result in less hypotension (measured in TWA) postoperatively at the Post Anesthesia Care Unit (PACU).

**Fig. 5:**
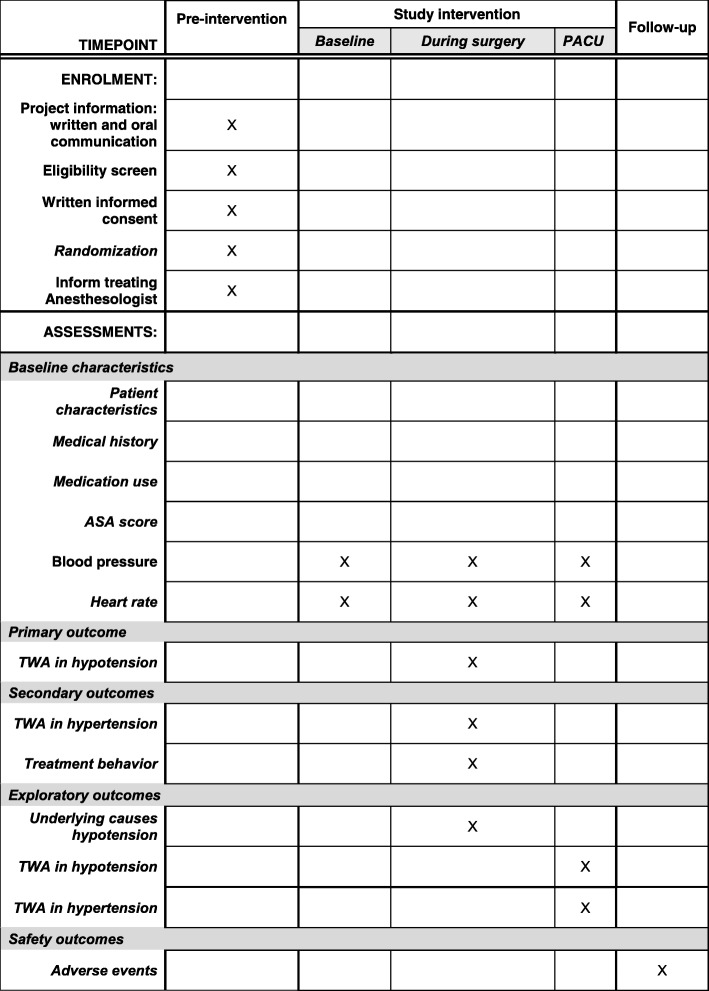
Schedule of enrollment, interventions and assessments. *PACU* Post Anesthesia Care Unit, *ASA* American Society of Anesthesiologists, *TWA* time-weighted average

For an overview of the outcome assessments see Fig. 5.

#### Safety

All adverse and serious adverse events, irrespective of causality, will be collected and reviewed by the principal investigator and reported to the Medical Ethics Committee of the AMC Amsterdam. Adverse events are defined as any undesirable experience occurring to a subject during the study, whether or not considered related to the experimental intervention. All adverse events reported spontaneously by the subject or observed by the investigator or his staff will be recorded.

Serious adverse events are defined as any untoward medical occurrence or effect that: results in death; is life-threatening (at the time of the event); requires hospitalization or prolongation of existing inpatients’ hospitalization; results in persistent or significant disability or incapacity; is a congenital anomaly or birth defect; or any other important medical event that did not result in any of the outcomes listed above due to medical or surgical intervention but could have been based upon appropriate judgment by the investigator. An elective hospital admission will not be considered as a serious adverse event.

We cover the potential harm of overtreatment by assessing the cumulative treatment dose during surgery and by assessing the amount of hypertension (in TWA, AUC, incidence, total time and percentage of time spent in hypertension). We will compare the outcomes between the control and intervention groups.

Insurance is provided for all participating subjects by the AMC Amsterdam.

#### Sample size calculation (phase B)

Difference in primary outcome will be compared using the Student’s *t* test or the Mann-Whitney *U* test, based on normality. A statistician performed the sample-size analysis. Based on previously published blood pressure data during surgery, it was estimated that our control group would have a TWA of 0.50 and a difference of 0.38 or larger between two study groups would be clinically relevant [18]. An effect size of 0.74 was calculated by dividing the estimated difference of 0.38 (mean experimental group − mean control group) by the standard deviation of 0.51. A sample size of 30 in each group in the randomized phase will have 80% power to detect an effect size of 0.74 using a two-group *t* test with a 0.05 two-sided significance level. Sample size was calculated using R 2017 [19].

The baseline data collection enables us to calculate the normal TWA spent in hypotension in our hospital and will be used to verify our sample-size analysis.

Patients who are randomized but in whom no study measurement was started, no arterial line was placed or when technical failure of the HemoSphere device prevented data collection will be excluded and replaced.

### Statistical analyses

We will analyze the data based on an intention-to-treat principle. The intention-to-treat population is defined as all patients who meet the inclusion criteria at the end of the study period.

Continuous data will be presented as median with range and/or interquartile range (IQR), or mean with standard deviation and range when normally distributed. Normality of distribution will be assessed visually with histograms and Q-Q plots. Categorical data were given as frequencies with percentages. For each of the analyses a probability value of *p* < 0.05 will be considered statistically significant.

Our primary outcome is TWA in hypotension (phase B). We will compare the TWA of each arm using the Student’s *t* test or Mann-Whitney *U* test, depending on the distribution of the data. The baseline data collection enables us to calculate the normal TWA spent in hypotension in our hospital and will be used to verify the representativeness of our control group. We will compare the TWA in the baseline group (phase A) to the TWA in the control arm (phase B).

The secondary and exploratory research questions involving categorical data will be analyzed using the *χ*^2^ test/Fisher’s exact test and secondary research questions involving continuous (numerical) data will be analyzed using the Student’s *t* test or the Mann-Whitney *U* test. Feasibility of working with HPI will be analyzed using qualitative research methods, reporting the number of protocol violations with reasons. Underlying causes of intraoperative hypotension will be analyzed using our study flowchart (Fig. 2) on all 100 patients. To assess whether use of HPI intraoperatively results in less postoperative hypotension at the PACU the TWA in hypotension during PACU stay will be analyzed. The exploratory questions will not be addressed in the primary article. All analyses for the primary article will be done using Matlab (R2018b) and SPSS (version 25).

### Monitoring

In accordance with the decision of our Medical Ethics Committee this trial is scored “low risk” and will, therefore, not need to be monitored by a Data Monitoring Committee.

### Ethical approval and registration

This study protocol has been approved by the Medical Ethics Committee of the AMC in Amsterdam. All protocol amendments will be communicated to the Medical Ethics Committee. The study protocol is in adherence with the Declaration of Helsinki and the guideline of Good Clinical Practice. Written informed consent will be obtained by trained researchers the day prior to surgery. A subject-screening and enrollment log will be kept on a secure server only accessible to study personnel. This trial has been registered with the NIH, U.S. National Library of Medicine at ClinicalTrials.gov (NCT03376347).

## Discussion

### Definition of intraoperative hypotension

Intraoperative hypotension is clearly associated with adverse postoperative outcomes [11]. Controversially, a universally accepted definition of intraoperative hypotension does not yet exist [2]. In this study, we define hypotension as a MAP below 65 mmHg which is in line with some large clinical trials and with our hospital’s protocol [14].

### Treatment behavior

For a machine-learning algorithm-based tool to help prevent intraoperative hypotension the treating anesthesiologists need to be willing to change their treatment behavior from reactive to proactive. Furthermore, the anesthesiologists will need to get used to diagnosing the underlying cause of hypotension based on the extra hemodynamic variables.

### Clinical relevance

The algorithm was developed using continuously measured waveform data from 1334 patients, internally validated on a cohort of 350 patients and externally validated on a cohort of 204 patients [15]. This is the first randomized controlled trial using this algorithm intraoperatively. This trial is powered on the TWA in hypotension. If this trial is successful in reducing intraoperative hypotension, future studies are needed and they will need to be powered to anticipated changes in clinical outcomes.

## Trial status

The study started with inclusion of the first patient in November 2017, the planned duration of the trial is 18 months. Last amendment of protocol: version 4.0 March 2018.

## Additional files

Additional file 1: Standard Protocol Items: Recommendations for Interventional Trials (SPIRIT) 2013 Checklist. (DOC 119 kb)

## Data Availability

The full protocol, dataset and statistical analysis plan will be available upon request to the corresponding author.

## Abbreviations

AAT: Area above the threshold
AMC: Academic Medical Center
ASA: American Society of Anesthesiologists
AUT: Area under the threshold
GDFT: Goal Directed Fluid Therapy
HPI: Hypotension Prediction Index
IQR: Interquartile range
MAP: Mean arterial pressure
PACU: Post Anesthesia Care Unit
TWA: Time-weighted average
